# The utility of human two plus one small pronucleated zygotes (2.1PN) based on clinical outcomes and the focused ploidy analysis

**DOI:** 10.1007/s10815-024-03114-9

**Published:** 2024-04-13

**Authors:** Hiromitsu Hattori, Noriyuki Okuyama, Kyota Ashikawa, Yoshiyuki Sakuraba, Hideki Igarashi, Koichi Kyono

**Affiliations:** 1 Kyono ART Clinic Sendai, 1-1-1 3F, Honcho, Aobaku, Sendai, Miyagi 980-0014 Japan; 2 Kyono ART Clinic Takanawa, Takanawa Court 5F, 3-13-1, Takanawa Tokyo, Minatoku 108-0074 Japan; 3 Kyono ART Clinic Morioka, 3F, 15-5, Moriokaekimaedori, Morioka-Shi, Iwate 020-0034 Japan; 4 HOPE (Human Ovarian-Tissue Preservation Enterprise), 4F 1-8-12 Shinagawa-Ku, Kitashinagawa, 140-0001 Japan; 5 Varinos Inc, DiverCity Tokyo Office Tower 21F, 1-1-20 Aomi, Koutou-Ku, Tokyo, Japan

**Keywords:** Embryo development, Embryo haplotyping, Live birth, Preimplantation genetic testing, Two plus one small pronucleated zygotes (2.1PN)

## Abstract

**Purpose:**

Are human embryos arising from two plus one small pronucleated zygotes, called 2.1 pronuclei (PN), clinically useful?

**Methods:**

In a retrospective embryo cohort study and prospective experimental study, a total of 287 cycles in which at least one 2.1PN was identified in the fertilization check were included. Embryonic development and clinical outcome were compared for the 1395 2PN zygotes and 304 2.1PN zygotes that were siblings. All embryos were individually cultured in time-lapse systems. Twenty-five 2.1PN-derived blastocysts, donated for research, were used in focused single-nucleotide variant ploidy analysis to identify the distribution pattern of heterozygosity.

**Results:**

The average diameter of PN was 24.9 ± 2.4 µm for large PN and 10.2 ± 2.4 µm for small PN; 79.9% of small PN was derived from female pronuclei. Blastocyst formation rate and good-quality blastocyst rate were significantly lower with 2.1PN embryos than with 2PN embryos (40.0% vs. 57.7%, 21.4% vs. 33.5%, respectively). A total of 13 embryos derived from 2.1PN were transferred, and three healthy babies were born. In ploidy constitutions of trophectoderm (TE), 2.1PN-derived blastocyst TE was shown to be mostly diploid (95.8%, 23/24), and only one blastocyst showed triploid.

**Conclusions:**

It was suggested that 2.1PN embryos have lower embryonic developmental potential than 2PN embryos, but most of the 2.1PN were diploid, indicating that they are likely to be clinically usable. It is recommended to perform embryo transfer following a combination of PGT-A and ploidy analysis.

**Supplementary Information:**

The online version contains supplementary material available at 10.1007/s10815-024-03114-9.

## Introduction

Human in vitro fertilization (IVF), either conventional IVF or intracytoplasmic sperm injection (ICSI), is performed after oocyte retrieval, followed by confirmation of fertilization by checking the number and shape of pronuclei (PN) and the extrusion of the second polar body 16–18 h later [1]. Today, many IVF laboratories perform fertilization assessments using a time-lapse incubator. The time-lapse incubator enables accurate evaluation of pronuclear numbers without missing the disappearance of PN due to syngamy through continuous observation over time.

A zygote with two even-sized PN is considered normal fertilization, while a zygote with one, three, or more than three PN is considered abnormal fertilization and is discarded in most cases. This is because embryos derived from abnormal fertilization zygotes are more likely to harbor abnormal haploid or polyploid chromosomal constitution (i.e., triploidy or tetraploidy), and the resulting risk of implantation failure, miscarriage, or hydatidiform mole is very high when these embryos are transferred [2]. On the other hand, several studies have reported successful pregnancies and live births following embryo transfer using 1PN or 3PN-derived embryos, suggesting that not all of these embryos necessarily have abnormal ploidy [3, 4]. Recently, the ploidy of 2.1PN embryos, which have two normal-sized PN with one additional small pronucleus, such as 3PN embryos, has shown that most of these embryos are diploid [5, 6]. A few papers have analyzed the embryonic development and ploidy of 2.1PN embryos and discussed their clinical usefulness, but the number of subjects in each case is small and their characteristics are still unknown [5, 6]. Furthermore, clinical results of embryo transfer have only been shown in three to four 2.1PN embryos [5, 7]. It is difficult to evaluate ploidy based solely on the morphology and number of PN, and 2.1PN embryos are often discarded as 3PN embryos are.

Conventional comprehensive chromosome testing (CCT) to analyze embryonic aneuploidy has limitations in accurately detecting abnormal ploidy, and embryos showing a euploid profile can still be tetraploid, triploid, or haploid. To evaluate the ploidy status, single-nucleotide polymorphisms (SNPs) and single-nucleotide variants (SNVs) are considered useful [5, 8].

In this study, we analyzed in detail the characteristics of 2.1PN zygotes using time-lapse imaging and evaluated their developmental potential and clinical outcomes compared to 2PN-derived embryos in a sibling-oocyte study. Furthermore, we aimed to explore the clinical utility of 2.1PN-derived embryos by assessing their chromosomal characteristics through aneuploidy analysis using CCT and abnormal ploidy analysis using SNV genotyping.

## Materials and methods

We conducted a retrospective cohort study of the developmental potential and clinical outcomes of 2.1PN embryos from August 2018 to July 2022 at Kyono ART Clinic. This study involved a total of 287 cycles in which at least one 2.1PN zygote was found at the time of the fertilization check. A total of 1395 2PN and 304 2.1PN zygotes were evaluated for embryonic development and clinical outcomes in the sibling embryo study. To avoid bias in clinical outcomes, the study does not include preimplantation genetic testing for aneuploidy (PGT-A) cycles.

**Fig. 1:**
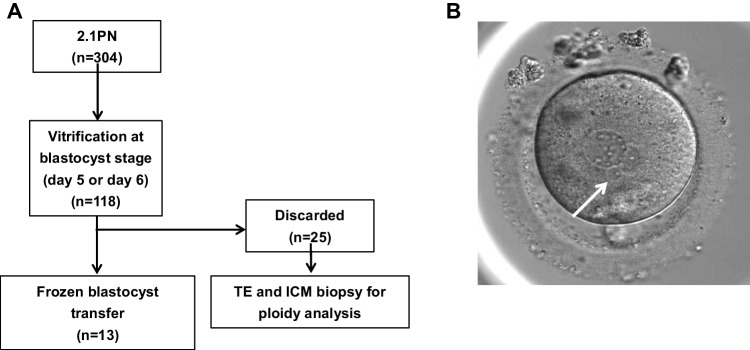
Study design flow chart and two plus one small pronucleated zygotes (2.1PN). (**A**) Allocation flowchart for the embryo transfer and ploidy analysis of 2.1PN-derived embryos. (**B**) 2.1PN was defined as two evenly-sized large PN and one small PN with single nucleoli (white arrow)

In addition, twenty-five 2.1PN-derived blastocysts, which were discarded at the patient’s request and donated for research, were subjected to biopsy and ploidy analysis as a prospective experimental study (Fig. 1A). This study was approved by the Institutional Review Board of Kyono ART Clinic on 30th July 2019 (reference number: 4102–190730). All procedures followed were in accordance with the ethical standards of the responsible committee on human experimentation (institutional and national) and with the Helsinki Declaration of 1964 and its later amendments. All the patients involved in this study have allowed us to use their medical record data for research in an unidentifiable manner.

### Ovarian stimulation

Ovarian stimulation was performed mainly with a combination of gonadotropin-releasing hormone (GnRH) agonist, follicular stimulating hormone (FSH), and human menopausal gonadotropin (hMG) or GnRH antagonist, FSH, and hMG. An injection of 5000–10,000 IU of human chorionic gonadotropin (hCG) was administered when the diameter of the dominant follicle reached 18 mm. Individual stimulation protocols were determined by patient age, FSH level at the start of ovarian stimulation, and anti-Müllerian hormone (AMH) level. Transvaginal ultrasound-guided oocyte retrieval was performed 36 h after hCG and/or GnRH agonist injection.

### Fertilization check and embryo culture

Insemination was conducted by standard in vitro fertilization (IVF) or ICSI according to the clinical indication. In IVF cycles, short insemination was performed and cumulus cells were removed 5 h after insemination [9]. All inseminated oocytes were individually cultured in time-lapse monitoring systems (TLM) (EmbryoScope + , Embryo Slide; Vitrolife, Denmark), including the oocytes which were immature after IVF, at 37 °C in a 6.0% CO2, 5.0% O2, and 89.0% N2 atmosphere. One step medium (CSCM-NX; FUJIFILM Irvine Scientific, Japan, and global® total®; CooperSurgical, USA) was used for embryo culture, and Embryo Slides were covered with mineral oil (HiGROW OIL Heavy; Fuso Pharmaceutical Industries, Japan). Zygotes showing 2PN were considered normal fertilization. 2.1PN was defined as two normal-sized PN with one additional small pronucleus that is not larger than half the size of normal and has only one nucleolus precursor body (Fig. 1B). The number and morphology of second polar bodies (PB) were also observed. Most embryos were cultured until days 5–6 and vitrified on reaching the blastocyst stage. The grading assessment of the blastocyst was performed based on the Istanbul Consensus [2]. The blastocyst vitrification and thawing procedures were undertaken according to the protocols of the Vitrification Kit (Kitazato, Japan) by using CryoTop (Kitazato, Japan) and Thawing Kit (Kitazato, Japan), respectively. Frozen-thawed blastocyst transfer was performed after the uterus was primed with estrogen and progesterone for luteal phase support. Frozen-thawed blastocyst transfer was preferentially performed on 2PN-derived blastocysts, and 2.1PN-derived blastocysts were transferred when no other 2PN-derived blastocysts were available. The physician provided a sufficient explanation of the possible risks to the patient, and the embryo transfer was performed after obtaining a general consent form. All consent forms are kept in the medical record.

### Measurement of PN diameter and assessment of the female and male origin of small PN by TLM

The diameters of the large and small PN were measured manually using the measurement tool attached to EmbryoViewer®. The measurement was performed when the pronuclear diameter was at its maximum. The female and male origin of the small pronucleus was determined based on the appearance pattern of the large pronucleus [10]. That is, when the small pronucleus was found near a large pronucleus emerging from the vicinity of the second polar body, it was assumed to be of female pronuclear origin, and when it was found near a large pronucleus emerging from near the center of the oocyte cytoplasm, it was assumed to be of male pronuclear origin. However, if the male and female PN appeared from the same position, they were considered of unknown origin.

### Ploidy assessment using SNV genotyping

Blastocyst biopsy was performed on 25 embryos arising from 2.1PN that were not used for embryo transfer and discarded at the patient’s request to determine ploidy. In 10 of the 25 embryos, only TE was analyzed (for technical reasons of biopsy), and in 15, both TE and ICM were analyzed. The biopsy protocol for ICM from blastocysts was performed based on that previously described [11]. Chromosome analysis and ploidy determination of TE and ICM were performed by SNV genotyping with next-generation sequencing (NGS). For chromosome copy number analysis, TE and ICM biopsy samples were processed using the VeriSeq PGS kit (Illumina, Inc., San Diego, CA, USA). For ploidy analysis, we analyzed all chromosomes by a multiplex polymerase chain reaction–based target sequence method. Reference allele and alternative allele of each SNP were counted, and the B allele frequency was determined for each sample to determine the ploidy. B allele frequencies were plotted for each sample, and diploidy was determined when there was a single heterozygous cluster. However, if two hetero clusters were identified, the sample was determined to be triploid.

### Data analysis

All statistical analyses were performed using JMP Pro13.1.0 (SAS Institute, Cary, USA). The Mann–Whitney *U* test was used to compare the pronuclear diameter of 2.1PN and 2PN embryos, and Fisher’s exact test was used to compare embryonic development and clinical outcomes of 2.1PN and 2PN embryos. We calculated the adjusted odds ratio (AOR) using multivariable logistic regression analyses to evaluate the association between the IVF protocol and the frequency of 2.1PN. The adjusted variables included IVF protocols, i.e., insemination methods (IVF or ICSI), sperm origin for ICSI (ejaculated or testicular), and oocyte status (fresh or frozen). These factors were selected since they could be regarded as potentially confounding factors. A two-sided *P* < 0.05 was regarded as statistically significant.

## Results

### Embryonic and clinical outcome of 2.1PN embryos

Table 1 shows the background data on the subjects. Of the 304 2.1PN embryos, 62 were derived from IVF and 242 from ICSI. No difference was observed in the second PB status of 2.1PN and 2PN embryos. The average diameter of two normal-sized PN in 2.1PN embryos was 24.9 ± 2.4 µm, which was significantly smaller than 25.2 ± 2.2 µm in 2PN embryos (*P* < 0.05). The average diameter of small PN in 2.1PN embryos was 10.2 ± 2.1 µm (Table 1). We analyzed the origin of small PN using TLM and found that most of them were derived from female PN (79.9%, 243/304), small PN originating from male PN were very rare (1.0%, 3/304), and unknown origin was observed in 19.1% (58/304) of cases (Supplementary Fig. 1).

**Table 1:** Characteristics of the study subjects, embryological and clinical outcomes of 2.1PN and 2PN-derived embryos

	2.1PN	2PN	P
Cycles	287		–
Maternal age (year)	39.3 ± 4.6		–
Paternal age (year)	40.7 ± 6.4		–
Maternal BMI (kg/m^2^)	22.0 ± 3.3		–
AMH level (ng/ml)	2.4 ± 2.7		–
Cause of infertility			
Ovarian reserve factor, *n* (%)	103/287 (35.9%)		–
Uterine factor, *n* (%)	31/287 (10.8%)		–
Tubal factor, *n* (%)	26/287 (9.1%)		–
Male factor, *n* (%)	57/287 (19.9%)		–
Unexplained, *n* (%)	38/287 (13.2%)		–
Other, *n* (%)	32/287 (11.1%)		–
AFC (*n*)	5.2 ± 2.6		–
Initial dose of gonadotrophins (IU/ml)	252.4 ± 59.1		–
Total dose of gonadotrophins (IU/ml)	2036.8 ± 799.4		–
Duration of stimulation (days)	8.8 ± 2.2		–
Estradiol levels at hCG-trigger	1153.5 ± 828.3		–
Mean no. retrieved oocytes	8.1 ± 5.7		–
No. of inseminated oocytes	2196 (IVF 633, ICSI 1563)		–
IVF	62/633	365/633	
ICSI	242/1563	1030/1563	
Total	304/2196	1395/2196	–
Second polar body status (%)			
2 PB	281/304 (92.4%)	1305/1395 (93.5%)	0.449
3 PB	1/304 (0.3%)	6/1395 (0.4%)	1.000
Fragmented	22/304 (7.2%)	84/1395 (6.0%)	0.433
Normal diameter PN (µm)	24.9 ± 2.4	25.2 ± 2.2	0.040
Small diameter PN (µm)	10.2 ± 2.1	–	–
Abnormal cleavage (%)			
Direct uneven cleavage	33/304 (10.9%)	153/1395 (11.0%)	1.000
Reverse cleavage	2/304 (0.7%)	20/1395 (1.4%)	0.404
Blastocyst formation (%)	118/295 (40.0%)	775/1342 (57.7%)	< 0.01
Good-quality blastocyst (%)	63/295 (21.4%)	450/1342 (33.5%)	< 0.01
Clinical pregnancy/FBT	4/13 (30.8%)	129/304 (42.4%)	0.568
Miscarriage/FBT	1/4 (25.0%)	37/129 (28.7%)	1.000
Live birth/FBT	3/13 (23.1%)	92/304 (30.3%)	0.761

*PN*, pronuclei; *BMI*, body mass index; *AMH*, anti-Müllerian hormone; *AFC*, antral follicle count; *hCG*, human chorionic gonadotrophin; *IVF*, in vitro fertilization; *ICSI*, intracytoplasmic sperm injection; *PB*, polar body; *FBT*, frozen-thawed blastocyst transfer

Data are shown as mean ± SD

As a result of evaluating the embryonic development of 2.1PN embryos, the blastocyst development rate was 40.0% (118/295), which was significantly lower than the 57.7% (775/1,342) of 2PN embryos (*P* < 0.05). Furthermore, the good blastocyst rate (ICM and TE grade 1 or 2) for 2.1PN embryos was 21.4%, significantly lower than the 33.5% for 2PN embryos (*P* < 0.05). Analysis of the incidence of abnormal cleavage identified in TLM showed no difference between the two groups for direct cleavage (division of one blastomere dividing into three cells) and reverse cleavage (blastomere fusion). We compared the clinical results of frozen-thawed blastocyst transfer and found no significant differences in pregnancy rates, miscarriage rates, or live birth rates between 2.1PN and 2PN embryos (Table 1). No congenital anomalies were found in the three babies derived from 2.1PN embryos.

Multivariate logistic regression analysis showed that there was no significant association between IVF protocols such as ICSI (AOR, 1.20; 95% CI, 0.87 to 1.64), use of testicular sperm (AOR, 0.76; 95% CI, 0.29 to 1.98), and use of frozen oocytes (AOR, 0.53; 95% CI, 0.24 to 1.17) and the incidence of 2.1PN (Table 2).

**Table 2:** Adjusted odds ratios of 2.1PN zygotes by method of IVF protocols

Insemination methods	No. of 2.1PN	AOR (95%CI)	P
IVF	62/633 (9.8%)	1.00(Ref)	
ICSI	242/1563 (15.5%)	1.20 (0.87–1.64)	0.264
Sperm origin for ICSI			
Ejaculated	237/1513 (19.2%)	1.00(Ref)	
TESE	5/50 (13.9%)	0.76 (0.29–1.98)	0.569
Oocytes			
Fresh	298/1650 (18.1%)	1.00(Ref)	
Frozen	6/49 (12.2%)	0.53 (0.24–1.17)	0.117

*PN*, pronuclei; *IVF*, in vitro fertilization; *ICSI*, intracytoplasmic sperm injection; *TESE*, testicular sperm extraction; *AOR*, adjusted odds ratio; *CI*, confidence interval

Adjusted for insemination method (IVF or ICSI), sperm origin for ICSI (ejaculated or testicular), and oocyte status (fresh or frozen)

### Ploidy data of 2.1PN by SNV analysis

Detailed data of 2.1PN embryos (*n* = 25) subjected to ploidy analysis are presented in Table 3. In the results of the ploidy analysis of TE, one embryo was undetectable, but most of the other 24 embryos were diploid (95.8%, 23/24), and only one embryo (4.2%,1/24) was observed to be triploid. Of the 15 embryos analyzed for ICM, all 13 embryos that were available for analysis were diploid (100%, 13/13) (Table 3). The diameter of the small PN of the triploid embryo was 15.0 µm, which was larger than the average diameter of the other diploid embryos (9.8 ± 1.9 µm). For embryos derived from 2.1PN, the timing of appearance and breakdown of large PN and small PN were analyzed in diploid and triploid embryos. The appearance time of small PN was faster in triploid than in diploid embryos (6.9 vs. 10.9 ± 2.4 h, respectively) (Supplementary Table 1).

**Table 3:** Embryologic and genetic data of the blastocysts derived from 2.1PN zygote

Sample ID	Female age (y)	Insemination method	Diameter of small PN	Blastocyst grade	Day of biopsy	Aneuploidy of TE	Aneuploidy of ICM	Ploidy of TE	Ploidy of ICM
1	34	ICSI	8	6AB	6	Euploid	–	Diploid	–
2	41	ICSI	12	5BC	5	Aneuploid	–	Diploid	–
3	40	ICSI	11	5BC	5	Aneuploid	–	Diploid	–
4	39	ICSI	9	5BC	6	Euploid	–	Diploid	–
5	43	ICSI	11	6AB	6	Euploid	–	Diploid	–
6	42	ICSI	10	5BB	6	Aneuploid	–	Diploid	–
7	40	ICSI	12	5BB	6	Aneuploid	–	Diploid	–
8	39	ICSI	12	5AB	7	Aneuploid	–	Diploid	–
9	41	ICSI	15	5BB	5	Aneuploid	–	Triploid	–
10	39	ICSI	11	5BA	6	Aneuploid	–	Diploid	–
11	39	ICSI	11	4BC	6	Euploid	Aneuploid	ND	Diploid
12	36	ICSI	6	4AB	5	Mosaic	Euploid	Diploid	ND
13	41	ICSI	11	4BB	6	Aneuploid	Aneuploid	Diploid	Diploid
14	44	ICSI	11	4BC	6	Aneuploid	Aneuploid	Diploid	Diploid
15	39	IVF	6	6BB	6	Aneuploid	Aneuploid	Diploid	Diploid
16	33	IVF	12	6BC	6	Euploid	Euploid	Diploid	Diploid
17	38	ICSI	7	6AB	5	Mosaic	Euploid	Diploid	Diploid
18	38	ICSI	12	6BC	5	Aneuploid	Aneuploid	Diploid	Diploid
19	37	IVF	11	4BC	5	Mosaic	Mosaic	Diploid	Diploid
20	38	ICSI	9	4BB	5	Mosaic	Mosaic	Diploid	Diploid
21	36	ICSI	8	4BB	5	Aneuploid	Aneuploid	Diploid	Diploid
22	36	ICSI	8	4BA	5	Aneuploid	Aneuploid	Diploid	Diploid
23	39	ICSI	9	4BC	5	Aneuploid	Aneuploid	Diploid	ND
24	44	ICSI	10	4BC	6	Aneuploid	Aneuploid	Diploid	Diploid
25	40	ICSI	8	4BB	5	Aneuploid	Aneuploid	Diploid	Diploid

*PN*, pronuclei; *IVF*, in vitro fertilization; *ICSI*, intracytoplasmic sperm injection; *TE*, trophectoderm; *ICM*, inner cell mass; *ND*, not detected

## Discussion

Using SNV-based ploidy analysis, we have demonstrated the characteristics of 2.1PN embryos presenting a small pronucleated nucleus. They were as follows. (1) Assessment of the PN using TLC suggested that 80% of small PN were derived from the female pronucleus. (2) The embryonic development rate of 2.1PN embryos was lower than that of 2PN embryos. (3) The embryos that would previously have been classified as 3PN may contain diploids if they have a small pronucleus that is not larger than half the size of normal and has only one nucleolus precursor body. These results suggested new characteristics of 2.1PN embryos. Because of the risks associated with the transfer of triploid embryos, which have been associated with miscarriage, they are generally excluded from embryo transfer. However, there have been reports of live births from 3PN-derived embryos, and there are many unknowns regarding the number of PN and chromosomal karyotypes [4, 12].

Although Capalbo et al. define the small pronucleus as less than 1/3 of the large pronucleus, in this study, diploidy was observed even in embryos with a 12-µm pronucleus, about half the size of the large pronucleus [5]. The small pronucleus in the 2.1PN with triploidy was 15 µm, slightly larger than in the other 2.1PN embryos. However, this is the result of only one embryo and should be taken with caution. In the present study, small PN was defined as not larger than half the size of normal and has only one nucleolus precursor body, and most embryos were diploid in SNV diploidy tests. This result could be a reference to determine ploidy by the size of the pronucleus, but further validation is needed because the size difference is very slight and the number of analyses is small.

2.1PN embryos showed a significantly lower blastocyst development rate and good blastocyst rate than sibling 2PN embryos. This result differs from a previous report that showed no difference in embryonic development between 2.1PN and 2PN embryos [6]. This may be due to the very limited number of 2.1PN in the previous report and the fact that the controls were not sibling study embryos. This study is a sibling-oocyte study in the same patients, and the number of fertilized oocytes was large. In general, it has been reported that the embryonic development of 1PN and 3PN embryos is lower compared to 2PN embryos because a higher percentage of 1PN and 3PN embryos have ploidy abnormalities such as haploid and triploid, which arrest before blastulation [13]. The reason for the reduced embryonic development was unclear because a higher percentage of 2.1PN embryos had diploids. Furthermore, the full morphokinetic data of 2.1PN embryos from the TLM showed no increase in abnormal cleavage patterns such as direct cleavage and reverse cleavage.

Analysis of the causes of 2.1PN showed no difference in the incidence between ICSI and IVF. The main causes of 3PN in IVF and ICSI are polyspermic fertilization and polar body extrusion failure [14, 15]. In the present study, embryos with three large PN were excluded from the 2.1PN subjects, and the second PB status of 2.1PN and 2PN embryos was similar. No association was found between ejaculated or testicular-derived sperm and the occurrence of 2.1PN, although it has been reported that spermatozoa in cases of male factors such as oligozoospermia and azoospermia have a higher proportion of polyploidy abnormalities [16, 17]. In addition, the use of frozen oocytes, which affect calcium oscillation during fertilization, was not associated with the occurrence of 2.1PN.

Detailed analysis of the TLC images revealed that approximately 80% of the small PN appeared near the female pronucleus, suggesting that the small PN was derived from the oocyte. It has been suggested that the occurrence of 2.1PN embryos is associated with maternal aging [6]. Oocyte chromosome aberrations also increase with age, but the relationship between chromosomal aberrations and 2.1PN is unclear. One hypothesis is that a phenomenon similar to trisomy rescue, which is rarely observed during somatic cell division, may occur during the pronucleation of 2.1PN embryos. However, examination of the chromosome karyotypes of the 2.1PN embryos revealed that only a few were mosaic and most were aneuploid. In the PGT-A analysis with TE biopsy, the euploid rate of 2.1PN embryos was 20.0% (5/25). This is slightly lower than the 25.5% shown in the results of a nationwide study by the Japan Society of Obstetrics and Gynecology [18]. The mean maternal age in both studies was almost the same at 39 years, and it is expected that maternal age is not the only factor contributing to the low euploid rate in 2.1PN embryos. Further studies are needed to determine the effect of 2.1PN on chromosome karyotypes.

In this study, we performed NGS analysis combined with SNV typing. This method allowed the simultaneous analysis of aneuploidy and abnormal ploidy. In an experimental analysis of the 25 embryos derived from 2.1PN, diploidy was confirmed in 24 embryos (96.0%) at TE or ICM. All euploid 2.1PN embryos were diploid. This SNV typing method is useful for the analysis of embryos with pronuclear numbers other than 2PN. Embryos derived from 1PN have been reported to be moles even though they were euploid, and most of the embryos derived from 3PN are triploid [12, 19]. By applying SNV typing to the analysis of these embryos, it may be possible to utilize 1PN and 3PN embryos which would previously have been discarded while reducing the risk of moles and miscarriages.

A strength of our study is the use of data from a single ART facility. There is no bias due to differences in laboratory procedures or culture protocols. In addition, because more cases were included in this study than in the previous report, and the culture results were obtained through a sibling-oocyte study, the influence of individual cases is considered to be low. However, there are some limitations to our study. First, the number of 2.1PN embryos used for embryo transfer was small. There were no significant differences in pregnancy, miscarriage, or birth rates between 2PN and 2.1PN embryos, but their effects on these rates are unknown. In addition, the number of embryos subjected to ploidy testing was also limited, and the relationship between small PN size and ploidy status remains unknown. Second, because this is a sibling-oocyte study, the influence of case characteristics on the occurrence of 2.1PN is unknown. Takahashi et al. have suggested that the incidence of 2.1PN is higher with advanced maternal age [6].

In conclusion, our results suggest that 2.1PN embryos have lower embryonic developmental potential than 2PN embryos. In the ploidy analysis, most of the 2.1PN were diploid, indicating that they are likely to be clinically usable. However, since triploid embryos were also observed, it is recommended to perform embryo transfer following a combination of PGT-A and ploidy analysis.

### Supplementary Information

Below is the link to the electronic supplementary material.

Supplementary file1 (PDF 181 KB)

Supplementary file2 (XLSX 10 KB)

## Data Availability

The data sets used and/or analyzed during the present study are available from the corresponding author upon reasonable request.
