# Supplementary material for: The utility of human two plus one small pronucleated zygotes (2.1PN) based on clinical outcomes and the focused ploidy analysis
Source: J Assist Reprod Genet. 2024 Apr 13;41(6):1589–96. doi: 10.1007/s10815-024-03114-9 (PMC11224203; doi:10.1007/s10815-024-03114-9)

**The utility of human two plus one small pronucleated zygotes (2.1PN) based on clinical outcomes and the focused ploidy analysis.**

Journal of Assisted Reproduction and Genetics

Hiromitsu Hattori, Noriyuki Okuyama, Kyota Ashikawa, Yoshiyuki Sakuraba, Hideki Igarashi, Koichi Kyono*
Corresponding author: Koichi Kyono
Kyono ART Clinic Takanawa, Takanawa Court 5F, 3-13-1, Takanawa, Minatoku, Tokyo, 108-0074, Japan
Email: kyono@ivf-kyono.or.jp

# Supplemental Figure 1

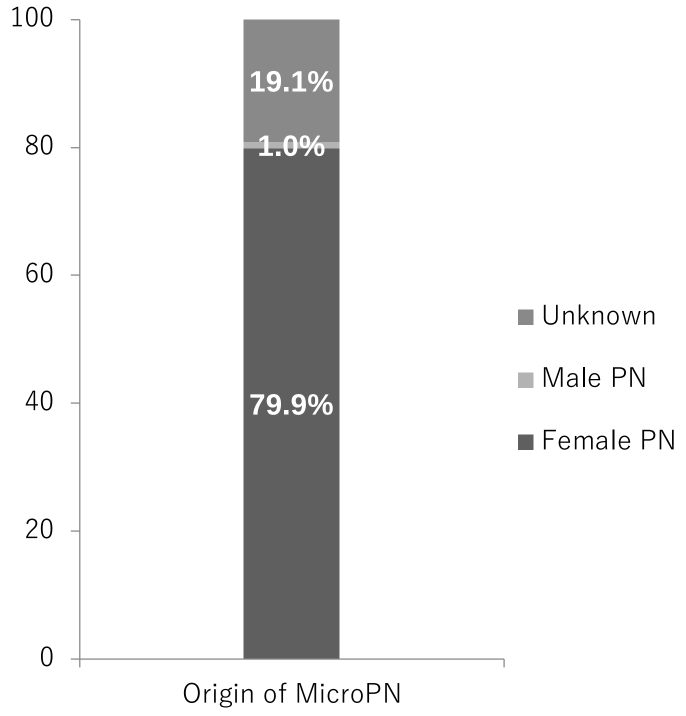

Supplement: Supplementary file 1 — Supplementary file1 (PDF 181 KB) [file 10815_2024_3114_MOESM1_ESM.pdf]
